# OG716: Designing a fit-for-purpose lantibiotic for the treatment of *Clostridium difficile* infections

**DOI:** 10.1371/journal.pone.0197467

**Published:** 2018-06-12

**Authors:** Johan A. Kers, Anthony W. DeFusco, Jae H. Park, Jin Xu, Mark E. Pulse, William J. Weiss, Martin Handfield

**Affiliations:** 1 Intrexon Corp., Industrial Products Division, South San Francisco, CA, United States of America; 2 Oragenics, Inc, Alachua, FL, United States of America; 3 University of Massachusetts Lowell, One University Avenue, Lowell, MA, United States of America; 4 PreClinical Services, UNT System College of Pharmacy, Fort Worth, TX, United States of America; Purdue University, UNITED STATES

## Abstract

Lantibiotics continue to offer an untapped pipeline for the development of novel antibiotics. We report here the discovery of a novel lantibiotic for the treatment of *C*. *difficile* infection (CDI). The leads were selected from a library of over 300 multiple substitution variants of the lantibiotic Mutacin 1140 (MU1140). Top performers were selected based on testing for superior potency, solubility, manufacturability, and physicochemical and/or metabolic stability in biologically-relevant systems. The best performers *in vitro* were further evaluated orally in the Golden Syrian hamster model of CDAD. *In vivo* testing ultimately identified OG716 as the lead compound, which conferred 100% survival and no relapse at 3 weeks post infection. MU1140-derived variants are particularly attractive for further clinical development considering their novel mechanism of action.

## Introduction

*C*. *difficile* continues to be an urgent threat in the hospital setting according to the Centers for Disease Control and Prevention (CDC), and one of the most common healthcare-associated infections worldwide, causing up to 250,000 infections per year, 14,000 deaths and resulting in at least 1 billion dollars in excess medical costs per year in the United States alone [1]. The most recent UK data available are that the 30-day mortality after diagnosis of CDI is approximately 16% and approximately half of those patients appear to die of CDI directly [2]. Deaths related to CDI increased 400% between 2000 and 2007, in part because of the emergence of a more virulent strain. Resistance to the antibiotics used to initially treat CDI (metronidazole and vancomycin) is not yet a significant problem, but may contribute to the development of resistance to vancomycin in other bacterial species. CDI mostly occur in people who have had both recent medical care and who were treated with antibiotics, and more frequently in hospitalized or recently hospitalized patients [2]. Recurrence is one of the major hallmarks for hospitalized CDI and approximately a quarter of patients will experience a recurrent episode [2]. While the epidemiology can markedly vary from country to country, many countries are seeing high current rates of CDI and rates generally appear to be increasing. The USA currently has very high rates that have been increasing in the last few years. In the UK, CDI remains an issue but the rates have, for a considerable number of years now, been declining. Over the last 2–3 years, the rates have plateaued and the most recent data available suggest a small increase in CDI rates in the UK of approximately 6%; that was the year 2014/2015 when compared with the previous 12 months [2]. New therapies are becoming available and include new drugs (e.g. fidaxomicin and rifaximin), the antitoxin antibody bezlotoxumab and other novel strategies including fecal transplantation (FMT), microbial restoration, phage therapy, *etc* [3, 4]. While new options for infectious disease treatment have been welcomed, there is concern that their high cost may prevent wide-spread use [5]. Nevertheless, there continues to be extensive research efforts to develop additional therapeutic alternatives due to the high unmet clinical need.

**Fig 1:**
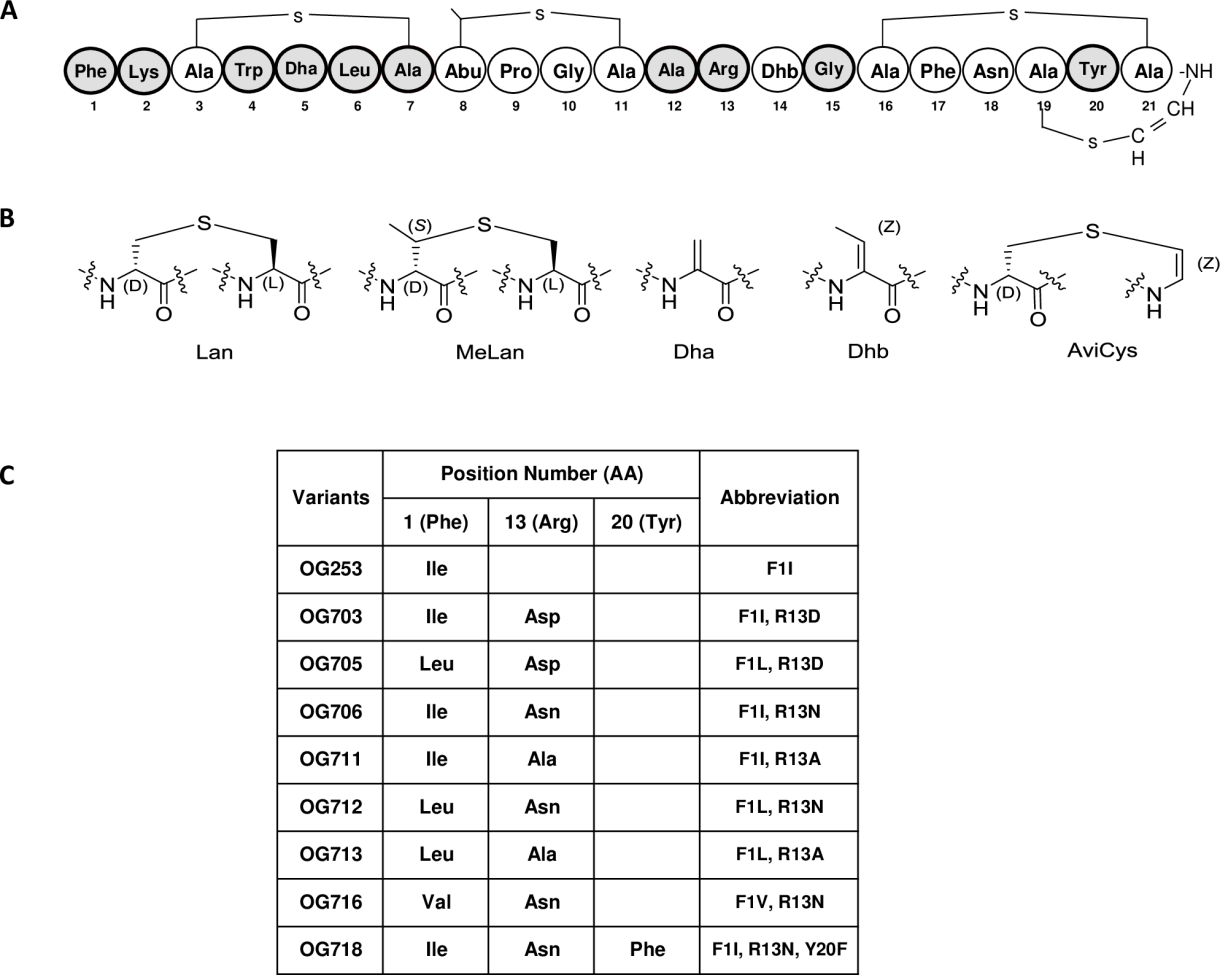
Structural features of MU1140 and select lead compounds. Panel (A) depicts the primary amino acid sequence of MU1140. The second generation of MU1140 variants designed in the current study focused on the residues highlighted in gray. Panel (B) depicts the structure of unusual amino acids. Panel (C) tabulates the substitutions of lead compounds carried through *in vivo* efficacy studies. Legend: amino acids, AA.

Lantibiotics are polycyclic lanthipeptides that have demonstrated antimicrobial properties. Nisin, which remains a paradigm for much of lantibiotic research [6, 7]. As shown in Fig 1, lantibiotics derive their names from the thioether ring containing amino acids lanthionine (Lan, Ala-S-Ala) and/or 3-methyl-lanthionine (MeLan, Abu-S-Ala). Lantibiotics often incorporate post-translationally modified amino acids such as 2,3-didehydroalanine (Dha), 2,3-didehydrobutyrine (Dhb), and the unsaturated lanthionine derivatives aminovinyl-*D*-cysteine (AviCys) at their *C*-terminus [8–10]. There is considerable evidence that indicates that lantibiotics may be efficacious and well tolerated as therapeutic agents in humans [7, 9–13]. Nevertheless, only a few lantibiotics have advanced into clinical trials. For example, the lantibiotic NVB-333 (Cantab Anti-infectives, UK) and NAI-107 (Naicons, Italy) continue to be in pre-clinical testing for treatment of Gram-positive bacterial infections, and NVB-302 (Novacta, UK) formally completed Phase-I clinical testing. The lanthipeptide duramycin did complete Phase-II studies, but although it has antimicrobial properties, it was developed for its ability to also stimulate chloride secretion through calcium-activated chloride channels in the context of treatment for cystic fibrosis [12, 14, 15].

Dr. Jeffrey D. Hillman’s group and his collaborators must be credited for the discovery and much of the seminal work performed on Mutacin 1140 (MU1140), a lantibiotic produced by the Gram-positive bacterium *Streptococcus mutans* [16, 17]. Today, the genes involved in the production and export of MU1140 have been identified and characterized [17, 18]. We also know its high-resolution structure as resolved by nuclear magnetic resonance (NMR) [19]. Years were spent in developing processes to produce and purify sufficient quantities of this compound with a sufficient degree of purity to explore its pharmacological properties. Today, we have a better understanding of its potency and spectrum of activity, including a low detected frequency for the development of antimicrobial resistance [20]. We also found that this class of molecules is relatively non-cytotoxic and possesses a good overall pharmacological profile [20, 21, 22]. One of the most exciting characteristics of MU1140, nisin and other closely-related molecules is that they act via a novel mechanism of action termed Lipid-II abduction [23, 24]. This unusual mechanism of action is thought to improve antibiotic durability due to the inherent difficulty in development of antibiotic resistance via modification of the pyrophosphate moiety of its target, Lipid-II [20, 23]. Lipid II is an essential constituent of cell wall synthesis. It is well known that several other antibiotics, such as vancomycin, target Lipid II at the terminal part of the pentapeptide [25]. Through the heavy natural selection resulting from broad use of vancomycin, bacteria have selected for mutation of the pentapeptide of Lipid-II without negatively affecting cell wall synthesis. A well characterized example of this phenomenon is the acquisition of *van*A by vancomycin-resistant enterococci, leading to the selection of vancomycin-resistant strains that effectively substituted the *D*-Ala-*D*-Ala of the pentapeptide of Lipid-II by a *D*-Ala-*D*-Lactate moiety [26]. In contrast, MU1140 and closely related molecules bind the pyrophosphate moiety of Lipid-II. This property may confer a lower frequency of resistance development than vancomycin because of the invariant ancestral nature of Lipid-II, the lack of mutational resistance due to amino acid substitution, and the inability of bacteria to develop resistance mutations without affecting peptidoglycan synthesis. It is interesting to note that nisin has been used in the food industry for over 60 years and in over 50 countries without leading to an overt development and spread of resistance or cross-resistance to other lantibiotics across microbial species of the gastrointestinal tract. Resistance/tolerance phenotypes against lantibiotics of therapeutic interest have been mostly obtained in the laboratory in order to investigate this phenomenon [27].

In recent years, MU1140 has become an extremely attractive scaffold to develop novel lantibiotics. In previous reports, we have gained insight into the potential pitfalls related to the production, purification and formulation of MU1140-related compounds [22, 28]. These issues have delayed the further development and clinical testing of MU1140 for several indications. We recently reported the engineering of a saturation mutagenesis library to study the contribution of each amino acid of the core peptide of MU1140 to the activity profile in an unbiased and addressable fashion [28]. In this work, 418 different single amino acid variants of MU1140 (22 positions x 19 possible permutations) were effectively created, screened and further characterized. The best performers from the saturation mutagenesis library for the treatment of *C*. *difficile* infections were identified based on: a) physicochemical properties, b) potency, c) spectrum of activity, d) stability and solubility in bio-relevant fluids (FaSSGF, FaSSIF and serum), and e) efficacy *in vivo* [22, 28].

Based on experience from earlier studies, we sought to pyramid the most productive amino acid substitutions into a second generation of MU1140-derived compounds in order to identify improved pharmacological properties, targeting CDI as a first indication. While previously identified compounds were efficacious *in vitro* and *in vivo* [22], they were prone to proteolytic degradation in gastrointestinal fluid systems and required additional formulation development work to assure enteric delivery and efficacy *in vivo*. These limitations were addressed in the current report and resulted in the identification of OG716, a lead compound that can be administered orally without any detected loss of efficacy relative to the performance of compounds delivered enterically. In particular, *in vivo* studies confirmed a 100% survival rate and the absence of relapse in a *C*. *difficile* model of infection.

## Materials and methods

### Bacterial strains and library construction

The construction of the MU1140 variants strain library was described in detail in previous work [28], see S1 Table. Briefly, variants were constructed in *S*. *mutans* JH1140 [16] by allelic replacement where the native chromosomal *lan*A gene was replaced with *lan*A variants encoding codon substitutions. Splicing by Overlap Extension (SOE) PCR was used to construct DNA vectors for integration of *lan*A variants into the JH1140 chromosome using a selectable erythromycin resistance marker from pVA891[29]. Strains were routinely grown on TSYEX agar or broth containing 3 μg/mL erythromycin, and incubated in a candle jar for 3 days at 37°C. PCR and Sanger DNA sequencing was utilized to confirm replacement of the chromosomal copy of *lan*A with the *lan*A variant encoded on the integration vector. The lantibiotic producing strains used in this study were listed in Table 1 are designated as SMxxx. Their corresponding lantibiotics are designated as OGxxx (e.g., strain SM716 produces the lantibiotic OG716).

**Table 1:** Selected strains used in this study.

Name	Property, genotype or characteristics	Source or reference
***S*. *mutans***		
JH1140	MU1140 hyperproducing strain of *S*. *mutans* derived from JH1000	16
SM152	JH1140::Erm (intergenic *lan*A’-*lan*B) (producing MU1140)	28
SM253	SM152 producing OG253 (Phe1Ile)	28
SM702	SM152 producing OG702 (Phe1Ala-Arg13Ala)	This study
SM703	SM152 producing OG703 (Phe1Ile-Arg13DAsp)	This study
SM705	SM152 producing OG705 (Phe1Leu-Arg13Asp)	This study
SM707	SM152 producing OG707 (Phe1Ile-Arg13Asp-Gly15Ala)	This study
SM708	SM152 producing OG708 (Phe1Ile-Trp4Met-Arg13Ala)	This study
SM711	SM152 producing OG711 (Phe1Ile-Arg13Ala)	This study
SM712	SM152 producing OG712 (Phe1Leu-Arg13Asn)	This study
SM713	SM152 producing OG713 (Phe1Leu-Arg13Ala)	This study
SM716	SM152 producing OG716 (Phe1Val-Arg13Asn)	This study
SM718	SM152 producing OG718 (Phe1Ile-Arg13Asn-Tyr20Phe)	This study
SM719	SM152 producing OG719 (Phe1Ala-Arg13Gly)	This study
***C*. *difficile***		
ATCC 9689	Ribotype 001 Toxin A/B^+^, Binary Toxin^-^, toxinotype O	Eurofins
BAA-1805	Ribotype 027 (NAP1) hypervirulent strain, Toxin A/B^+^, Binary Toxin^+^, toxinotype IIIb	Eurofins
BAA-1875	Ribotype 078 (NAP7), Toxin A/B^+^, Binary Toxin^+^, REA BK 16, toxinotype V	Eurofins
BAA-1874	Ribotype 002 (NAP6), Toxin A/B^+^, Binary Toxin^-^, REA G1, toxinotype O	Eurofins
ATCC 43597	Ribotype 014, Toxin A/B^+^, Binary Toxin^-^, toxinotype O	Eurofins
BAA-1808	Ribotype 020, Toxin A/B^+^, Binary Toxin^-^, toxinotype O	Eurofins
UNT103-1	VA11, non epidemic (cdtB-, REA group J)	UNTHSC^1^
UNT107-1	Ribotype 027 (NAP1) hypervirulent strain, Toxin A/B^+^, Binary Toxin^+^	UNTHSC^1^
***M*. *luteus***		
ATCC 272	Reporter strain sensitive to Mutacins	ATCC

^1^ Received from Curtis Donskey, Cleveland VA Hospital, Cleveland, OH.

### High-throughput activity testing on *Micrococcus luteus*

A robotic high-throughput screening method was used to confirm the biological activity of each variant strain using *M*. *luteus* as a reporter strain (see experimental details in [28]). The activity of each compound was compared to the activity of OG253 (MU1140-Phe1Ile) in duplicate experiments. The choice of OG253 as a comparator and control was based on its superior performance in previous work [22]. As presented in S2 Table, scoring for activity was automated with an optical scanner [28], and the compounds were classified according to the size of their zones of inhibition (greater, equal to, or smaller than OG253).

### Compound manufacturing, and characterization by ultra performance liquid chromatography/mass spectrometry (UPLC/MS) and high performance liquid chromatography (HPLC)

Compounds tested in this study were produced by fermentation and purified by Intrexon Corp (1–10 mg scale) and Oragenics (100 mg scale). Detailed experimental conditions have been previously reported [28]. Briefly, 1 mg scale fermentations were performed in 1 L shake flasks, while 10–100 mg scale fermentations were performed in a Bioflo3000 (New Brunswick Scientific) 1-5L bioreactor using fed-batch fermentations under aerobic stirred-tank conditions with automated temperature/pH/dissolved oxygen controls. For small scale purifications (1–10 mg), culture supernatants were chloroform extracted and purified by reverse-phase flash chromatography (modified from [16]). Larger scale (~100 mg) purifications were carried out using column chromatography (Oragenics, unpublished). The purity and identity of each compound during manufacturing was determined by UPLC/MS as previously reported [28], data not shown). The purity and quantification of the compounds under solubility and stability testing was determined by HPLC as previously reported [28]. The stability in bio-relevant fluid was determined by LC/MS on Waters XBridge C18 column, particle size 3.5 μm, 4.6 x 70 mm. Buffer A was 0.1% formic acid in H_2_O and buffer B was 0.1% formic acid in acetonitrile (ACN). The gradient was 20% buffer B to 95% buffer B over 20 min at a flow rate of 0.4 mL/min. Injection volume was 25 μL. The mass of peak of interest was analyzed by electrospray ionization mass spectrometry with source temperature of 250°C and cone gas flow of 60 L/hr.

### Minimum inhibitory concentration (MIC) testing

MIC testing for *M*. *luteus* was performed using the broth dilution assay as described by the clinical and laboratory standards institute (CLSI) guidelines M07-A8 for MIC-testing of aerobic bacteria [30]. Briefly, *M*. *luteus* ATCC 272 was streaked onto a Luria-Bertani (LB) agar plate from a freezer stock and grown at 30°C for 48 hours. A single colony was transferred into 5 mL of LB broth in a 14 mL culture tube and grown overnight at 30°C in a shaker at 250 rpm. Cells were diluted 1:100 in 50 mL Mueller Hinton II broth in a 250 mL Erlenmeyer flask and grown shaking for 2 hr at 30°C, 200 rpm. *M*. *luteus* was transferred to a 50 mL screw cap tube, centrifuged for 5 min at 3220 x *g*, re-suspended in fresh Mueller Hinton II at an OD_600_ of 0.05, mixed by vortexing at high speed for 30 s, poured into a 50 mL sterile reservoir and inoculated into 96 well plates in 100 μL volumes using a 12 channel pipette. MU1140 and variants were serially diluted 2-fold in 50% ethanol, and 1 μL volumes were added to each well using a 12 channel pipette. Serial dilutions were performed starting at ~1 mg/mL for the highest concentration tested. Each test substance was evaluated in duplicate. The plates were shaken for 16 hr at 30°C and quantified using a plate reader to determine well OD_600_ values. OD_600_ values were normalized by wells containing medium only. MIC determinations were made following wells that contained OD_600_ ≤ 0.1 after 16 hr of growth.

MIC testing for *C*. *difficile* was performed by Eurofins Panlabs Taiwan LTD based on modifications from the CLSI M11-A8 broth dilution method for *Bacteroides fragilis* susceptibility testing [31]. Briefly, Reinforced Clostridial Medium (RCM) was pre-reduced by over-night incubation in an anaerobic chamber (80% N_2_, 10% CO_2,_ and 10% H_2_) prior to use. MU1140 and variants were dissolved in 100% DMSO, diluted by 2-fold serial dilution in 100% DMSO, for a total of 11 concentrations. A 4 μL aliquot of each dilution was added to 96 μL RCM in wells of a 96 well plate using a Biomek FX instrument (Beckman Coulter, USA), followed by the addition of *C*. *difficile* suspension, in RCM, 100 μL/well. The final bacterial count was 2 x 10^5^ to 1 x 10^6^ colony forming units/mL, in 200 μL per assay well. The test article concentration ranged from 64–0.0625 μg/ml, and the DMSO concentration was 2% in all wells. Assay plates were incubated at 35°C for 46 to 48 hr in the anaerobic chamber (A35 Anaerobic Workstation, Don Whitley, UK). Following incubation, test plates were visually examined and each well was scored for growth then the MIC was recorded as the lowest concentration that results in complete inhibition of growth. Each test substance was evaluated in duplicate. Vehicle-control and vancomycin were used as blank and positive controls, respectively. The MIC values of the vancomycin controls were consistent with the historical data against test strains (data not shown). The strains used in this study are presented in Table 1.

### Forced degradation

Five aliquots of each compound were prepared by dissolving them to 1.0 mg/mL in HPLC grade water (Fisher Scientific, USA). A total of 2 aliquots of the solution were placed in a 37°C stability chamber, and 2 aliquots were placed in a 50°C chamber. Samples were collected on days 0, 2 and 7, centrifuged at 12,000 x g for 5 min, and the supernatants were transferred to a fresh tube. Each sample was diluted 1:4 with 0.05% trifluoroacetic acid (TFA) and the purity and quantity were analyzed by HPLC as described above.

### Metabolic stability in fasted state simulated gastric fluid (FaSSGF) and fasted state simulated intestinal fluids (FaSSIF)

FaSSGF was supplemented with purified porcine pepsin (800–2500 U/mg) to a final concentration of 300 μg/mL, as previously described [22]. Samples were collected at 0, 2, 4, 8 and 24 hours. Compounds were quantified by LC-MS using the method described above. FaSSIF was supplemented with 2% pancreatin (w/v), 6 μg/mL of trypsin and 6 μg/mL of α-chymotrypsin and was prepared as previously reported [22]. All proteases were from Fisher ScientificSamples were collected at the same frequency as in the FaSSGF study, and analyzed using the same method.

### Formulation in 5% D-mannitol and solubility assessment

Testing the solubility of the test compounds in a commonly used excipient was a critical control, to assure that the formulation did not inadvertently precipitate the peptide as it is being administered to animals. To that end, approximately 10 mg of each compound was weighed on a high-precision balance and dispensed into a 1.5 mL microfuge tube. Samples were initially resuspended in a 5% *D*-mannitol (Sigma, USA) in water to 24 mg/mL, vortexted and visually assessed for solubility based on turbidity or the presence of insoluble particles. Samples were then sequentially diluted to 18 mg/mL and 12 mg/mL using 5% *D*-mannitol, and their solubility reassessed.

### Efficacy assessment

*In vivo* efficacy was performed as previously described in detail [22], except that the compounds were administered via oral gavage (rather than the illeal cannulation method used previously). This study was carried out in accordance with protocols 2016–0015 approved by the Institutional Animal Care and Use Committee (IACUC) at the University of North Texas Health Science Center (UNTHSC). IACUC established guidelines ensuring that approved protocols are in compliance with federal and state laws regarding animal care and use activity at UNTHSC. The UNTHSC animal program in USDA registered (74-R0081) and fully AAALAC accredited.

Briefly, 7–8 weeks old Male Golden Syrian hamsters were used in this study, and weighted 100–120 g (Charles River Laboratories, Wilmington, MA). *C*. *difficile* UNT103-1 (VA11, non epidemic (cdtB-, REA group J) was received from Curtis Donskey, Cleveland VA Hospital, Cleveland, OH) and cultured as previously described [28]. The isolate has been previously utilized for the hamster model by the University of North Texas Health Sciences Center (UNTHSC) [32]. The infection inoculum was tested to determine the ratio of spores to vegetative cells. In accordance with the details in Table 2, a total of 60 animals were used in this study. Animals were randomized into the 10 study groups (N = 6 per group) prior to treatment. On Day 0, animals were infected with with 5.63 x 10^7^ total CFU of which 2.81 x 10^4^ represented inoculated spores. On Day 1, at 24 hrs post-infection, all animals received a single subcutaneous injection of clindamycin (10 mg/kg). Test articles in 5% mannitol were then administered orally 3x per day (TID), starting on Day 2 at 18 hours after clindamycin injection, for 5 consecutive days (Days 2 through 6). USP-grade vancomycin (positive control) was prepared in water for injection (WFI) at 50 mg/mL and stored refrigerated during the course of the study. The vancomycin stock solution was diluted fresh daily to the appropriate concentration in WFI and administered at 20 mg/kg QD on Days 2 through 6 and the infection control group was administered test article vehicle in the same manner. All test articles and vehicle control were dosed with 0.19 mL volume. The identity and concentration of test articles were kept blinded during the study, and 5 individual aliquots of 4.1 mL/tube were provided frozen at ≤ -70°C. Each tube, containing sufficient volume for one full day of dosing 6 animals TID with ~20% excess, was thawed at the morning dose and kept refrigerated (5°C) between doses. After the final dose of the day, the remaining solution was returned to ≤ -70°C storage. Following completion of the study, all tubes were returned to the sponsor for testing, to confirm stability, purity and activity. Animals were observed three times a day for the duration of the experiment. The cecal contents from all hamsters that died on study or from hamsters euthanized by CO_2_ inhalation at the end of the observation period (Day 21) were collected and aliquoted into two samples. One sample was used for *C*. *difficile* toxin AB test (tgcBIOMICS GmbH, Bingen, Germany) and the other stored frozen at -80C for use in total (spore and CFU) viable counts. Data was analyzed using GraphPad Prism 6.0d. Survival was compared using Log-rank test, Gehan-Breslow-Wilcoxon test. Statistical significance was established at p ≤ 0.05.

**Table 2:** Hamster CDAD study design.

Group	Test Article(s)	Regimen	Route	Dose (mg/kg)	N =
1	**OG253 (F1I)**	TID x 5 days	Oral gavage	20	6
2	**OG703 (F1I-R13D)**			20	6
3	**OG706 (F1I-R13N)**			20	6
4	**OG711 (F1I-R13A)**			20	6
5	**OG712 (F1L-R13N)**			20	6
6	**OG713 (F1L-R13A)**			20	6
7	**OG716 (F1V-R13N)**			20	6
8	**OG718 (F1I-R13N-Y20F)**			20	6
9	Vancomycin	QD x 5 days		20	6
10	Infection (Vehicle) Control	N/A		N/A	6

## Results

### Triage strategy

**Fig 2:**
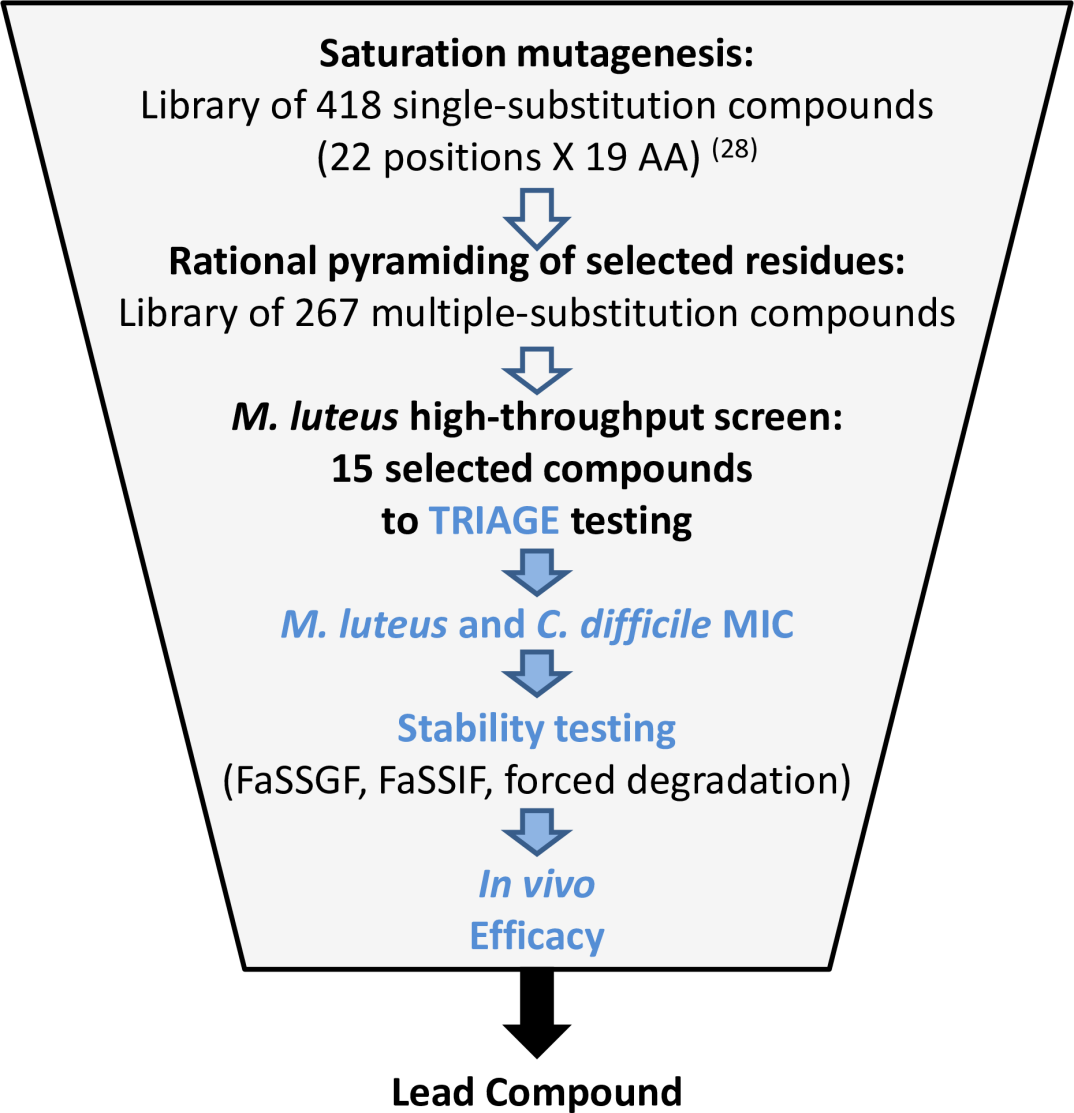
Triage strategy used in this study. High-throughput activity screening used robotic spotting and an optical scanner for the determination of the zones of inhibition. MIC testing provided specific activity values for the determination of the potency. Stability testing performed using biologically-relevant substrates. *In vivo* efficacy performed in the Golden Syrian hamster model of CDAD. See **Materials and Methods** section for details. Legend: FaSSGF, fasted-state simulated gastric fluid; FaSSIF, fasted-state simulated intestinal fluid.

The test compounds were engineered by pyramiding multiple amino acid substitutions in *lan*A, the primary structural gene encoding the pro-peptide that matures to form MU1140 (see S1 Table) based on performance improvements observed from single amino acid substitutions during previous studies [22, 28]. Each compound was produced at the mg-scale and its antimicrobial activity was tested by zone of inhibition on a high-throughput screening involving a reporter strain. The best 15 performers were produced at the 10 mg scale, purified to >90% purity by HPLC and subjected to a battery of tests designed to triage compounds for subsequent animal testing. The overall triage strategy is illustrated in Fig 2, which included a) *in vitro* potency assays, b) the characterization of the metabolic stability, and c) the characterization of stability by forced degradation. The top 8 performers from the *in vitro* assay screening process were produced at the 100 mg scale and tested against OG253 (MU1140-F1I) and vancomycin to evaluate their *in vivo* efficacy.

### Library construction and high-throughput activity screening

**Fig 3:**
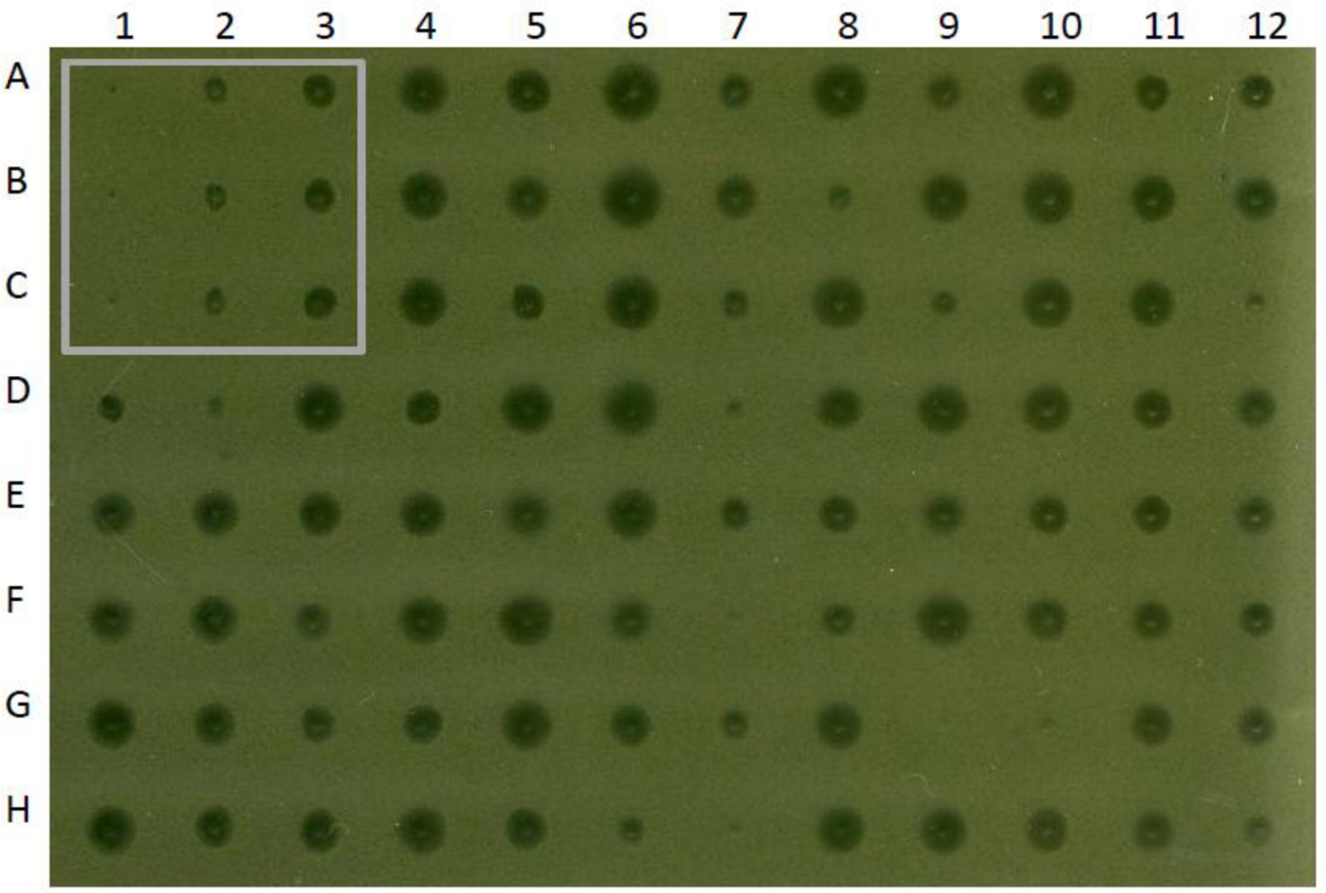
Illustration of the relative zones of inhibition of screened compounds versus MU1140 and OG253. Controls are boxed in the upper-left corner and include blanks (Column 1, negative control), MU1140 (Column 2, positive control) and OG253 (Column 3, comparator).

Of the 301 constructs initially designed, 267 were successfully constructed (see S1 Table for details), and their nucleotide sequences were confirmed by DNA sequencing (data not shown). The 34 unsuccessful constructs were assumed to fail because of technical and/or biological reasons (e.g, lower than anticipated transformation efficiency, pleiotropic effect, genetic reorganization, etc…). The initial ranking of the potency of compounds encoding multiple amino acid substitutions was based on the antimicrobial activity of *S*. *mutans* culture supernatants towards *M*. *luteus*, and is presented in S2 Table. A representative example of the data generated with this approach is depicted in Fig 3. Compounds with multiple substitutions generally outperformed OG253 (MU1140-F1I), as they generated larger zones of growth inhibition relative to OG253. A total of 155 compounds performed better than OG253 while 112 compounds performed equal or worse than OG253. Specifically, the data suggested that a) compounds with double substitutions that also included a F1I substitution outperformed OG253 83% of the time (10/12), b) substitutions that included a F1G outperformed OG253 80% of the time (8/10), c) compounds that included F1V substitutions resulted in greater activities compared to OG253 54.5% (6/11) of the time, and d) substitutions that included F1A substitutions performed better than OG253 50% (6/12) of the time. Interestingly, only compounds that included F1A, F1G, F1I, F1L, F1T, and F1V strains resulted in greater zones of inhibition (>0.319cm^2^ zone of clearing), as compared to OG253 (0.167cm^2^ zone of clearing). A total of 13 different amino acid substitutions were made at residue 13, and all of these substitutions were functional and performed better than or equal to OG253, in at least 1 permutation. R13A substitutions were over-represented in 7 of 32 total compounds and produced zones of clearing greater than 0.319cm^2^. In contrast, all compounds that incorporated a F1E (12/12) or F1P (9/9) substitution produced zones of inhibitions smaller or equal to OG253. Seventy-seven percent (7/9) of compounds with an F1N substitution performed equally to or were inferior to OG253. Based on these data and *M*. *luteus* high-throughput screening and experience from previous studies [22, 28], 15 compounds were selected for manufacturing at larger scale and additional testing.

### Characterization of manufactured compounds

The purity and identity of 15 purified compounds were produced in 10–100 mg scale and characterized by HPLC and LC-MS (data not shown). The purity of variants varied from 76–96% and the identity of the primary product peak was confirmed for each variant. No unexpected post-translationally dehydrated byproducts were observed. In contrast, the deletion of the amino acid residue at the *N*-terminus was observed for each compound that incorporated a Phe1Leu substitution. That the *N*-terminal Leu1 was either degraded or potentially incorrectly processed during export (representing 5–10% of the total amount produced) is a concern for each Phe1Leu containing compounds. Minor oxidation (+16 Da) and hydrolysis (+18 Da) byproducts were also observed during the manufacturing of OG711 and OG713, respectively, but to a lesser extent (<5% of the total amount of compound).

### *In vitro* potency against *C*. *difficile*

To assess the *in vitro* potency of each of the 15 selected variants of MU1140, their MIC was determined using a small panel of representative *C*. *difficile* strains (N = 8). *M*. *luteus* 272 was used as a control to assess the consistency with zones of inhibition testing, and previous reports [20, 28]. The complete MIC dataset is presented in S3 Table. In general, there was consistency between *M*. *luteus* and *C*. *difficile*, with the most potent compounds against the indicator strain also being the most potent compounds against *C*. *difficile*. Several compounds that were initially characterized by a larger zone of inhibition on *M*. *luteus*, as compared to OG253 (see Fig 3), demonstrated a lower relative potency by MIC testing with the same reporter strain upon repeat testing (see S3 Table). The MIC distribution of the top 8 performing compounds versus vancomycin and OG253 were analyzed, and the mode, range, MIC_50_ and MIC_90_ for *C*. *difficile* were calculated (see Table 3). These 8 compounds were evaluated in subsequent testing.

**Table 3:** MIC characteristics of selected compounds against *C*. *difficile*^1^.

Compounds	Range (μg/mL)	Mode (μg/mL)	MIC _50_ (μg/mL)	MIC _90_ (μg/mL)
**OG253 (F1I)**	0.06–0.5	0.25	0.25	0.5
**OG703 (F1I R13D)**	0.5–2	1	1	2
**OG705 (F1L R13D)**	0.5–2	1	1	2
**OG706 (F1I R13N)**	0.125–1	0.5	0.5	1
**OG711 (F1I R13A)**	0.25–1	0.25	0.5	1
**OG712 (F1L R13N)**	0.125–0.5	0.25	0.25	0.5
**OG713 (F1L R13A)**	0.25–0.5	0.5	0.5	0.5
**OG716 (F1V R13N)**	0.25–0.5	0.5	0.25	0.5
**OG718 (F1I R13N Y20F)**	0.25–1	0.5	0.5	1
**Vancomycin**	0.5–4	0.5	0.5	4

^1.^ n = 8

### Forced degradation

Forced degradation studies were used to evaluate the chemical stability of MU1140 variants. As shown in Table 4, stability by forced degradation was collected for up to 7 days and at 2 temperatures (37°C and 50°C). It was observed that most of the variants were stable at 37°C for 2 days. In contrast, three compounds (OG708, OG715 and OG718) showed signs of degradation by 7 days at 37°C (<20% recovery). Degradation was characterized by visual aggregation and insolubility. The compounds degraded faster at the higher temperature (50°C). Seven compounds showed signs of degradation and aggregation within 2 days (< 20% recovery), and most compounds, except OG702 (F1A R13A), showed signs of degradation and aggregation by day 7 at 50°C. Based on LCMS analysis (data not shown), common byproducts of degradation included mixtures of oxidation (+16 Da), hydrolysis (+18 Da) and deamidation (likely at residue Asn13 forming a succinimide, -17 Da).

**Table 4:** Solubility, stability under forced degradation, and half-life in bio-relevant matrices.

Compound	Forced degradation^1^								Solubility	Half-life^2^	
	2 Days at 37°C		7 Days at 37°C		2 Days at +50°C		7 Days at +50°C		5% *D*-mannitol (mg/mL)	FaSSGF (min)	FaSSIF (min)
	Δ Purity (%)	Δ Conc (mg/mL)	Δ Purity (%)	Δ Conc (mg/mL)	Δ Purity (%)	Δ Conc (mg/mL)	Δ Purity (%)	Δ Conc (mg/mL)			
OG253	-1.44	-18%	0.54	-14%	0.39	-14%	-3.65	-22%	≥ 24	>1440	14
OG703	-0.97	-9%	-1.04	-13%	-1.74	-15%	-4.79	-30%	7	>1440	606
OG705	-0.29	-11%	-0.61	-12%	-1.32	-11%	-3.22	-26%	ND	>1440	>720
OG706	-1.15	-11%	-6.64	-20%	-8.05	-23%	-21.46	-50%	18–24	>1440	708
OG711	-1.24	-13%	-0.63	-11%	-0.61	-9%	-2.94	-40%	12–18	>1440	>720
OG712	-0.21	-5%	-4.63	-9%	-3.01	-9%	-18.06	-45%	12	>1440	587
OG713	-0.06	-12%	-0.56	-9%	-1.51	-17%	-6.98	-41%	ND	>1440	577
OG716	-0.31	-7%	-5.20	-15%	-3.67	-17%	-19.77	-47%	12–18	>1440	552
OG718	-2.07	-9%	-7.42	-21%	-4.90	-29%	-12.84	-44%	≥ 24	>1440	>720

^1.^ Performed in UMASS Lowell

^2.^ Only the data from bio-relevant fluids supplemented with pepsin (FaSSGF) or pancreatin, trypsin and α-chymotrypsin (FaSSIF) is shown. The half-life in FaSSGF without protease supplementation was >1440 min. for all compounds, while the half-life in FaSSIF was >720 min. for all compounds, except for OG253 (422 min.) in the absence of protease supplementation.

### Metabolic stability

Metabolic stability studies are useful tools to predict the susceptibility of peptides to biotransformation in the context of drug selection with favorable pharmacokinetic properties. The physicochemical environment encountered during transit through the gastrointestinal (GI) tract is particularly harsh for therapeutic peptides. The relative stability of selected compounds was investigated in two biologically-relevant fluids: fasted-state simulated gastric fluid (FaSSGF) and fasted-state simulated intestinal fluid (FaSSIF). The contribution of proteolytic degradation was also assessed by addition of a cocktail of proteolytic enzymes from the GI tract to the bio-relevant fluids. As presented in Table 4, all compounds tested were stable in FaSSGF, with or without the addition of the proteolytic enzyme pepsin. In contrast, stability in FaSSIF was more variable amongst the compounds tested and the degradation pattern was markedly greater with supplementation with the proteolytic enzymes trypsin and chymotrypsin. The latter allowed ranking of different compounds based on their calculated half-life (Table 4). Interestingly, the inherent proteolytic sensitivity of OG253 to trypsin and chymotrypsin could be overcome in compounds that integrated an Ala, Asn or Asp substitution at Arg13. For example, OG253 (F1I) presented with a half life of ~14 min, while an additional R13A substitution in OG711 (F1I-R13A) extended the half life to >720 min, which represents a >50-fold improvement in stability to proteolytic degradation.

### Solubility in 5% *D*-mannitol

The solubility of the lead compounds in 5% *D*-mannitol was measured to characterize their basic properties in the formulation used for subsequent animal studies. As presented in Table 4, it was observed that a substitution of Arg13 (positively charged residue) with an Asn (non-charged but polar residue) resulted in a decrease in solubility. When Arg13 was substituted with Asp (a negatively charged amino acid), the solubility was further decreased, likely because of the progressive change of overall charge from +3 in OG253 (F1I) to +2 in OG706 (F1I-R13N) and to +1 in OG703 (F1I-R13D).

### Efficacy assessment *in vivo*

**Fig 4:**
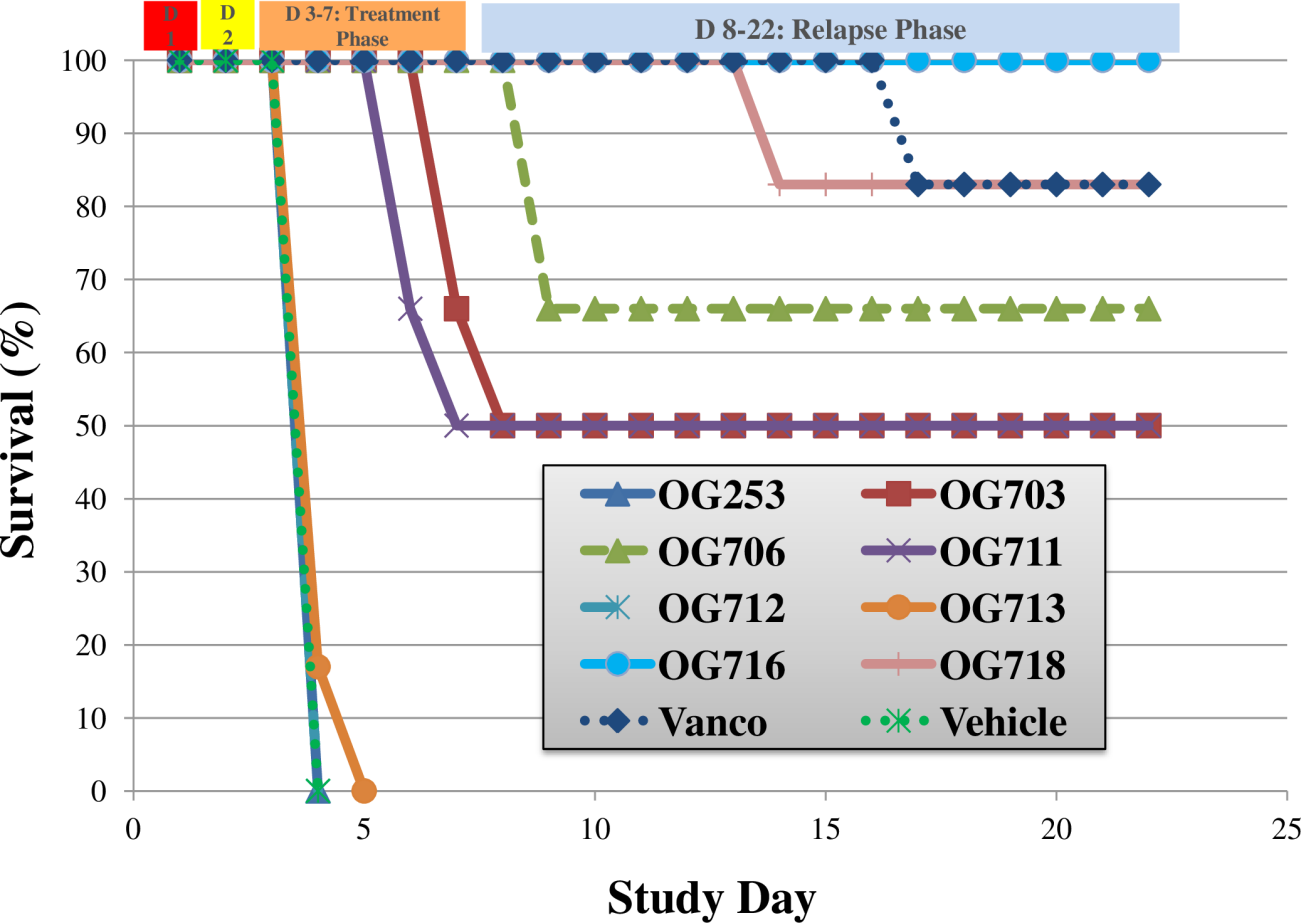
Efficacy of lead compounds assessed *in vivo*. Golden Syrian hamsters (N = 6 per group) were infected on Day 1 and received a single subcutaneous injection of Clindamycin (10 mg/Kg) on Day 2. Test compounds at 20 mg/Kg in 5% mannitol were administered by oral gavage 3 times per day (TID), starting on Day 2 at 18 hours after Clindamycin treatment, for 5 consecutive days (Days 2 through 6). Vancomycin (positive control) was administered at 20 mg/kg QD in parallel, and the infection control group was dosed with vehicle alone. See **Materials and Methods**for details.

**Fig 5:**
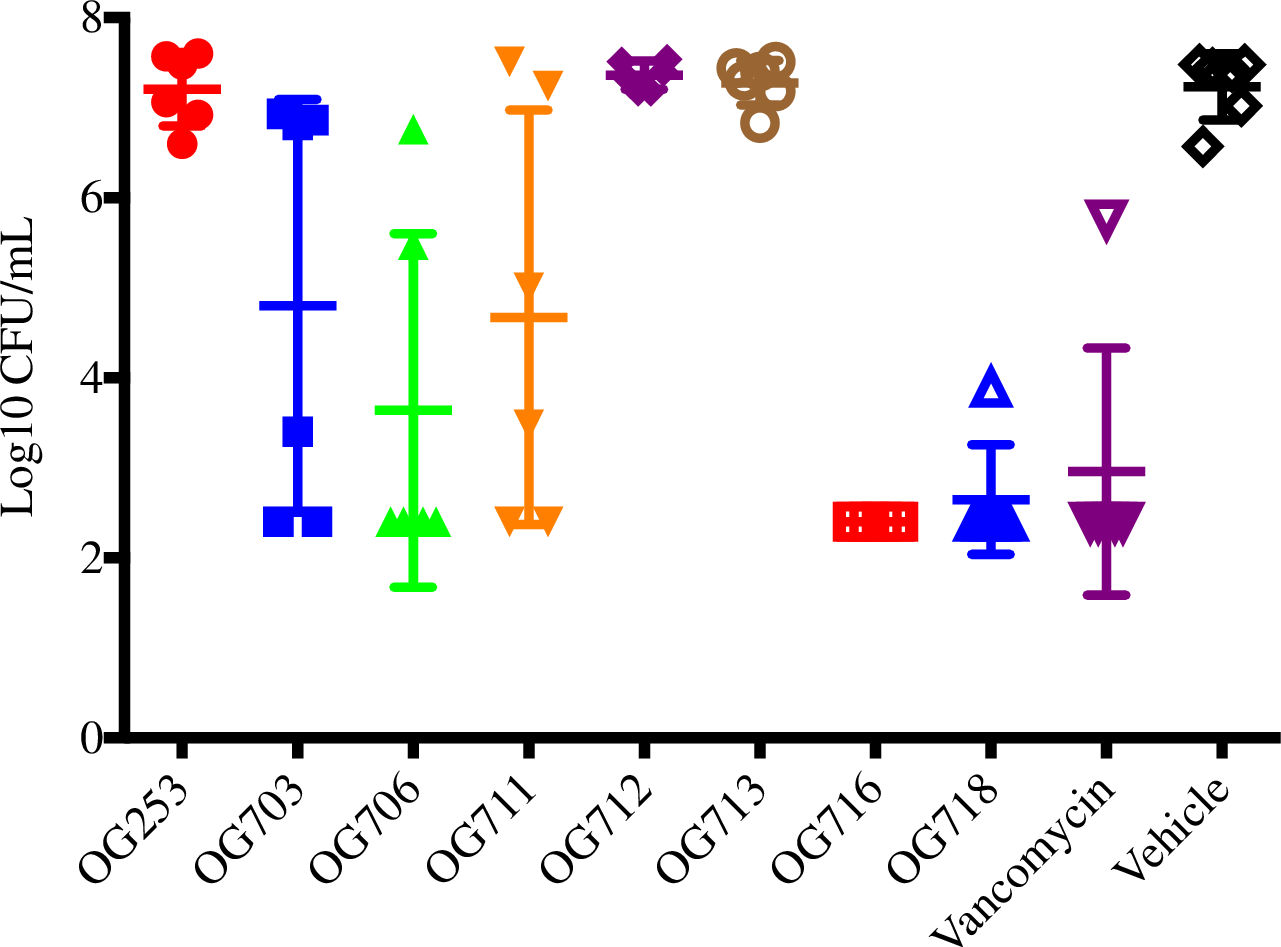
Spore titers at death or at the outcome of the treatment phase. The cecal contents from all hamsters that died on study or from hamsters euthanized at the end of the observation period (Day 21, see Fig 4) were collected and tested for spore counts (see **Materials and Methods** section for details).

As presented in Fig 4, the oral administration of several compounds resulted in enhanced survival of infected animals (overall survival or time to mortality) as compared to the vehicle control group. The lantibiotics OG703, OG706, OG711, OG716 and OG718 were significantly more efficacious as compared to the vehicle control group (Log-rank Mantel-Cox & Gehan-Breslow-Wilcoxon; *p*<0.05). Compounds OG253, OG712 and OG713 were no different (*p*>0.05) than the vehicle control group. Only the compound OG716 showed 100% survival at the outcome of the study, and was superior to vancomycin during the relapse phase (*p*<0.05). The survival difference between the two best performers, OG716 and OG718, was statistically different (*p*<0.05). The results for the *C*. *difficile* spore analysis of cecal samples for all animals on study are presented in Fig 5. OG716, which demonstrated 100% survival, exhibited spore counts near the lower limit of detection (2.40 log_10_ CFU/mL). The vancomycin treatment group (83% survival) had a mean spore count of 2.96 log_10_ CFU/mL. All other groups with survival rates from 50–83% exhibited spore CFU counts of 2.65–4.8 log_10_ CFU/mL. The spore counts for the OG716, OG718 and vancomycin-treated animals (p<0.0001) and for the OG711-treated animals (p<0.05) were significantly lower as compared to control (vehicle-treated) animals.

The results of the toxin analysis on collected cecal samples are presented in S1 Fig. Levels of Toxins A and B in the cecal contents tracked with both the measured CFU counts and survival during the study. Toxin A levels ranged from 26.3–8217 ng/mL (mean 3877 ng/mL) and Toxin B levels ranged from 32.1–3595 ng/mL (mean 1478 ng/mL) for animals that died on study. Surviving animal titers were below the limit of detection (≤ 0.8 ng/mL) for both toxins.

Overall, the OG716-treated group exhibited superior performance relative to all experimental compounds tested and compared favorably to the vancomycin group in all efficacy parameters (Fig 4). Vehicle control animals exhibited 100% mortality, corresponding to high *C*. *difficile* CFU and toxin titers while administration of 20 mg/kg OG716 TID (60 mg/kg/day) resulted in 100% survival (Fig 4) with bacterial CFU (Fig 5) and toxin titers (S1 Fig) at or below the limit of detection for each assay.

## Discussion

The large increases in the incidence and frequency of antibiotic resistance drives much of the search for new analogs of known antimicrobial compounds, new effective drug combinations as well as novel classes of antimicrobial compounds. Certainly, finding new compounds that exert their action via novel mechanisms in combination with more aggressive antibiotic stewardship programs in the clinic may offer opportunities to extend the life span of both approved and new antibiotics currently under development. In turn, extending the life span of new and currently available antibiotics may favorably impact the direct R&D and medical costs, as well as the indirect societal costs associated with inadequate treatment of infectious disease. As reviewed elsewhere, lantibiotics appear as increasingly attractive new scaffolds for the bio-engineering and chemical synthesis of new compounds that promise to be less susceptible to the development of resistance, based on their intrinsic properties [7, 10, 12, 15].

It was initially sought to identify specific residues of MU1140 that could modulate or even improve specific physico-chemical or pharmacological properties of the molecule, while preserving its distinctive therapeutic characteristics. To that end, a saturated single amino acid substitution library containing 418 compounds was constructed, which constituted an unbiased collection of single amino acid substitutions of MU1140. This library was then tested by focusing on the degree of potency, the toxicity profile, the stability and solubility of the compounds in different bio-relevant media [28]. It became apparent, from those studies that the naturally occurring Mutacin 1140 had several weaknesses that hindered its “drugability”. Importantly, these deficiencies could be addressed using a protein engineering strategy to either directly or indirectly improve the performance of MU1140 variants in a therapeutic context. Lantibiotics derived from MU1140 are amenable to many amino acid substitutions throughout the length of the core peptide, as long as the substitutions fall outside of the lanthionine residues in Rings A and C/D, Dhb14 and the AviCys22 precursor [28]. Several substitutions of the Phe1 residue were previously identified that could enhanced the half-life of MU1140 in bio-relevant fluids. Several substitutions of the Arg13 residue were also identified, which enhanced the half-life of MU1140 in simulated fluids by substitution by an amino acid residue that is not prone to proteolytic degradation [22, 28]. Those permissive substitutions affected, in certain instances, the intrinsic stability and solubility properties of the resulting molecules [28]. Ultimately, single amino acid substituted variants of MU1140 were shown to be efficacious *in vivo* as long as they were appropriately formulated and delivered directly to the colon [28]. While past work indicated that variants with improved potency would be challenging to discover based solely on single amino acid substitutions, markedly improved pharmacokinetic properties could indeed be engineered, and it was speculated that these MU1140 variants would likely need to combine several single substitutions to result in a lead candidate with a desired therapeutic profile [22]. The present report sought to pyramid multiple permissive amino acid substitutions in order to improve the physico-chemical and pharmacological properties of the resulting variant without affecting potency or manufacturability, and hence identify lead compounds for further drug development and human testing. Altogether, over 700 different single and multiple position mutated variants of MU1140 were engineered, which constitutes the largest library of any lantibiotic reported to date.

The current report pyramided 301 multiple amino acid substitutions based on previous observations from MU1140 variants containing a single amino acid substitution. Recognizing that increasing the half-life of the lanthipeptides was central to improve the pharmacologic properties of MU1140, over half of the substitutions made (156 total, see S1 Table) targeted double mutations in Phe1 and Arg13 only. Previous work had demonstrated that pyramiding does not necessarily lead to additive MU1140 variant performance improvement. For example, while both Trp4Ala and Arg13Asp single variants of MU1140 had increased potency on a deferred-antagonism assay, the double Trp4Ala & Arg13Asp variant resulted in reduced potency [33]. Consequently, Phe1 substitutions included amino acids predicted to confer a greater half-life (e.g., Val, Gly, Pro and Ile), but several additional amino acids were tested as well. Similarly, substitutions at Arg13 included ~13 residues per Phe1 residue in the matrix, attempting to provide a broad coverage of representative types of amino acids (positively and negatively charged, polar, hydrophobic and others), even including amino acids such as Lys, which is a known substrate for trypsin. This was performed in an attempt to generate unbiased structure-function information and uncover new or unexpected MU1140 variants that lead to performance improvements. In addition, to the double amino acid substitutions tested, up to eight amino acid substitutions were incorporated into individual MU1140 variants, in an attempt to add structural diversity and to include substitutions that presented advantageous properties that were discovered in previous work [28]. All permutations are presented in S1 Table and focused on selected additional substitutions in residues Lys2, Trp4, Ser5, Leu6, Ala12, Gly15 and Tyr20 of MU1140.

The evaluation of cytotoxicity and off-target pharmacological profiling are arguably key assays in the development of new drugs. Hepatotoxicity remains the most frequent reason cited for regulatory withdrawal of approved drugs [34]. Extremely low levels of cytotoxicity were previously observed at high concentrations with variants of MU1140 (mMolar range), which remains approximately 3 orders of magnitude higher than the expected therapeutic concentration (low μMolar to high nMolar) [22]. A thorough *in vitro* off target pharmacological profiling was previously performed on several variants of MU1140 using the Safety Screen 44^TM^ [35, 36], which suggested that this class of compounds was of relatively low overall toxicity and not likely to present inadvertent off-target effects [22]. That no obvious toxicity has been identified prior to *in vivo* evaluation is significant. However, because of their high degree of stability, it can be expected that engineered lantibiotics may accumulate in the kidney if they were delivered by the parenteral route, which may ultimately establish the dose limiting toxicity of this class of molecule.

The first step in the triage of the 301 multiple substitutions library was based on a high-throughput screen using a reporter strain. We have previously reported that while this methodology allows to rapidly screen through many variants and detect those with no or significantly reduced activity, this screen did not normalize the amount of compound tested in each spot, and as a result, only provided semi-quantitative data. Clearly, any substitution that would change the overall charge, polarity, hydrophobicity of a given variant may also affect its diffusion rate, stability and solubility, thereby confounding analysis [28]. Despite those limitations, it was noted that over half of the compounds tested performed better than the positive control used in this study, OG253 (MU1140-Phe1Ile, see [28]), and a great majority of compounds that included a Phe1Ile substitution outperformed OG253 (83%) as judged by their larger zones of inhibition.

Based on the high-throughput results and considering the lessons learned in previous studies [22, 28], 15 compounds were selected for further MIC testing using purified (>90% purity by HPLC) and quantified amounts of compound, on a clinically-relevant collection of *C*. *difficile* strains. Vancomycin was used as an additional control as a standard of care for *C*. *difficile* infection. Several compounds that were initially characterized by a larger zone of inhibition on *M*. *luteus*, as compared to OG253 (see Fig 3) actually demonstrated a lower relative potency by MIC testing with the same reporter strain and/or *C*. *difficile* (see S3 Table). The MIC profiles of the 8 most potent compounds against *C*. *difficile* strains (see Table 3) compared similarly to OG253 and favorably to vancomycin. Of particular interest, the MIC_50_ of 7 of the 8 compounds tested, and the MIC_90_ of every compound tested were superior to vancomycin by 2-fold to 8-fold (see Table 3), which remains somewhat surprising considering that those lantibiotics are relatively larger (~2.2 KDa) compared to vancomycin (~1.4 KDa) and target the same molecular target (Lipid II), albeit at a different moiety (pyrophosphate versus pentapeptide, respectively). Previous reports support potential use of MU1140 and mutants of enhanced potency against other microorganisms such as MRSA and VRE [20, 33]. The MIC profile of a larger sample size of microorganisms will be required to draw any significant conclusions regarding the potency of the MU1140 variants reported here against bacterial species other than *C*. *difficile*.

Forced degradation is an important part of the drug development process as it provides knowledge about the degradation chemistry of drug substances and drug products. This knowledge could be used for formulation or packaging development, and for the design of IND-enabling stability studies. The observation that the majority of variants were stable at 37°C for 2 days without any special excipient suggest that they can be relatively easily formulated for clinical use. At higher temperature (50°C), aggregation was observed, which led to insolubility. The identification of oxidation, hydrolysis and deamidation byproducts are common to all peptide therapeutics and generally mitigated during formulation by the addition of excipients that counteract this negative effect. None of the lead 8 compounds contained Ser or Thr, and consequently, no unexpected post-translationally dehydrated byproducts were observed [28]. However, the deletion of the first amino acid residue was observed for each compound that incorporated a Phe1Leu substitution. It remains unclear whether this is due to aberrant processing during secretion or the product of degradation in the culture liquor during fermentation or downstream processing.

Previously, the metabolic stability of single substitution MU1140 variants was reported to directly impact the pharmacokinetic properties of those compounds in the GI tract because of the sensitivity of Arg13 to proteolytic degradation [22]. We confirmed here that MU1140-related variants appear to be stable in FaSSGF, even with the supplementation with the proteolytic enzyme pepsin, as no degradation of MU1140 variants was detected. We also confirmed that a double substitution of residues Phe1 and Arg13 in MU1140 improves the half-live in FaSSIF (with trypsin/chymotrypsin) by a factor of no less than 39-fold, and up to >50-fold in certain instances. However, it does not appear that the half-life in any bio-relevant medium is, by itself, sufficient to predict and rank compounds’ efficacy *in vivo*.

The *in vivo* efficacy dataset supports the concept that a double substitution is sufficient to confer 100% hamster survival from CDI, as well as confer a sufficient degree of stability in the GI tract to be efficacious by oral gavage. In the hamster, the lead compound OG716 (Phe1Val-Arg13Asn) was superior to the other compounds tested. The lead was closely followed by all substitutions that contained an Ile in position 1, and included in order of efficacy OG718 (Phe1Ile-Arg13Asn-Tyr20Phe), OG706 (Phe1Ile-Arg13Asn), OG703 (Phe1Ile-Arg13Asp) and OG711 (Phe1Ile-Arg13Ala). All compounds containing a Phe1Leu substitution were outperformed by those containing a Phe1Val or Phe1Ile in this model, which was not expected considering that the only difference between Ile and Leu is the position of a -CH_3_ in the latter at the *N*-terminus of the compound. While we have previously reported that a Phe1Ile substitution was superior to other compounds tested in a cannulated hamster model of infection [22], we show here that a Phe1Val combined to an Arg13Asp or an Arg13Asn (with or without a Tyr20Phe) is superior in a non-cannulated oral gavage animal model. This finding reemphasizes the importance of eliminating the Arg13 proteolytic degradation site in MU1140.

Of particular relevance to a drug potentially targeting CDAD is the finding that the spore and Toxin A/B titers correlated with clinical outcomes. In fact, both the spore counts (≤ 2.4 Log CFU/g) and the toxin levels (≤ 0.8 ng/mL) were below the limit of detection in OG716-treated animals. Whether or not this compound has sporicidal properties, can inhibit germination or even directly modulate toxin levels as a function of time of exposure or dose dependence remains to be elucidated and is under investigation.

Solubility and formulation studies are often overlooked in early-stage drug discovery programs, but remain an integral part of drug development. A direct correlation between efficacy, solubility and stability was not found in the current study using a 5% *D*-mannitol formulation (in water). In fact, the lead compound (OG716) presented with an average solubility in this media, while the least soluble compound (OG703) was part of the top half of performers in animals. However, a correlation was confirmed between charge and solubility. When the positively charged Arg in OG706 (Phe1Ile-Arg13Asn) was substituted with a negatively charged Asp in OG703 (Phe1Ile-Arg13Asp), the overall charge of the compound was reduced from +2 to +1 and the solubility reduced 2-3-fold. The efficacy of OG706 (66% survival) was greater than OG703 (50% survival) at the outcome of the study, but the difference was not statistically significant (Log-rank test *p* = 0.3699, Wilcoxon test *p* = 0.2752).) It remains unclear whether or not the intrinsic solubility of this class of compounds at administration may be a relevant parameter to optimize the efficacy of the compound. By comparison, fidaxomycin is practically insoluble in water (0.0125 mg/mL, [37]), but effective in animals and humans. Perhaps it is ultimately a matter of assuring that the compound is appropriately dispersed to maximize the surface contact between the drug and its target. Dose-range finding studies may help resolve these issues by defining a minimal therapeutic threshold.

As an evolutionary response to their ecological niche, *C*. *difficile* and other Gram-positive organisms have naturally developed tolerance mechanisms against lantibiotics and other cationic antimicrobial peptides, as we have previously discussed [22]. These mechanisms include, for example, increasing the net positive charge of the cell wall or cell membrane, proteolytic degradation, sequestration, export through efflux pumps, the development of biofilms and immune mimicry (reviewed in [27]). While immunity to lantibiotics and tolerance mechanisms have been described for lantibiotic producing strains [38] and a few human pathogens [39], those levels of resistance remain below therapeutic ranges, in most instances. For example, the induction of the *cpr*ACB operon *in vitro* decreases the susceptibility levels of *C*. *difficile* against several cationic antimicrobial peptides [40, 41]. However, there remains no data available showing that these genes are induced *in vivo* or associated with the development of lantibiotic-resistance in humans at levels that would cause *C*. *difficile* to become refractory to therapy. For example, a modest 2–4 fold MIC increase of *C*. *difficile* to gallidermin to ~1 μg/ml levels [40] does not imply that a strain harboring this resistance phenotype would be refractory to gallidermin therapy. While mutants conferring an increased MIC may spontaneously arise at the *cpr* locus [40], there is no data available on the frequency of such mutation under biologically-relevant conditions, nor any data to support that these type of mutations would be selected for in the GI tract of mammals in a similar fashion as the vancomycin-resistant strains of enterococci that have emerged in the clinic [42]. It is noteworthy to reiterate that nisin resistance has not been correlated with modifications of Lipid II after over 50 years of use as a food preservative.

In summary, we describe in this report a new compound, OG716, a second-generation derivative of MU1140 engineered with multiple substitutions. OG716 emerged as the lead compound based on a better MIC profile than vancomycin, adequate properties in forced degradation, favorable half-life in biologically-relevant fluids and ultimately on its *in vivo* efficacy. Of particular clinical relevance, relapse was not observed at the conclusion of the *in vivo* study, consistent with the low spore and toxin levels detected. Collectively, the Phe1Val and Arg13Asn substitutions in OG716 address MU1140’s deficiencies as a lead candidate by conferring improved physico-chemical and pharmacological properties to this lead compound targeting at *C*. *difficile* infections. This is a particularly attractive proposal considering that these molecules exert antimicrobial activity by a novel mechanism of action that abducts Lipid II from the septum of division via the pyrophosphate moiety, leading to low anticipated levels of resistance development.

## Conclusions

Several lessons transpired from engineering and testing 418 compounds in the first-generation (single amino acid substitutions, see 28) and an additional 301 compounds in the second-generation (multiple amino acid substitutions) of MU1140-derived variants: 1) MU1140 is a “drugable” scaffold characterized by only a few invariant structural residues, enabling the pyramiding of beneficial multiple amino acid substitutions; 2) substitutions are not necessarily binomial (additive or subtractive); pyramiding requires using a sufficiently large collection of permutations to ultimately select for the compound with the desired properties; 3) high-throughput screening and *in vitro* testing are effective tools to study large combinatorial libraries of novel compounds, despite their innate limitations; 4) in instances where a particular pathogen cannot be used during the initial screening phases, the use of a surrogate reporter organism is adequate, but the data must be evaluated appropriately. Specific activity testing should be performed when feasible, as the determination of activity, using zones of inhibition, must be interpreted with care; a given substitution that modifies the overall charge, lipophilicity or hydrophobicity of a variant may also affect the diffusion rate, stability and solubility, potentially confounding the interpretation; 5) oxidation and hydrolysis byproducts are detected during manufacturing, but they only presented minor contaminants in the MU1140 variants tested; 6) the stability in the simulated gastric environment was high for all compounds tested (degradation not detected in the experimental assay) and the sensitivity of native MU1140 to intestinal proteolytic degradation could be abolished in several variants; and 7) the metabolic stability improvements engineered into OG716 enabled direct oral administration. Peptide drugs are generally administered via parenteral injection to circumvent problems related to poor bioavailability and/or lack of stability during transit through the GI tract [43]. We anticipate that the lessons drawn from MU1140 are generally applicable to other lanthipeptide therapeutics.

## Supporting information

S1 Table Multiple substitution matrix of MU1140 variants. The universal one-letter code is used. Numbers indicate the residue number relative to Mutacin 1140. Ser5 is dehydrated to a Dha5 during post-translational modification. Positively charged amino-acids in green (R, H and K); negatively charged amino-acids in blue (D and E); polar amino-acids in yellow (S, T, N and Q); hydrophobic amino-acid in red (A, V, I, L, M, F, Y and W). Failed substitutions highlighted in gray.
(PDF)

S2 Table High-throughput activity testing on *M*. *luteus*. Top eight (8) compounds are highlighted in red.
(PDF)

S3 Table Full characterization of top compounds selected from high-throughput screen. ^1.^ Highlighted in green indications that the value is lower than positve control, vancomycin.
^2.^ Highlighted in red indications that the Δ purity is lower than -10%.
^3.^ Highlighted in red indications that the Δ concentration is less than -20%.
^4.^ Highlighted in red indication that the solubility is below 10 mg/mL.
^5.^ Highlighted in red indication that the half life is below 300 min.
(PDF)

S1 Fig *C*. *difficile* cecal toxin titers on collected cecal samples. Levels of toxins A and B in the cecal contents tracked with both the measured CFU counts and survival during the study.
(PDF)

## Abbreviations

Abu: aminobutyric acid
AviCys: aminovinyl-*D*-cysteine
CDAD: *C*. *difficile* associated disease
CDI: *C*. *difficile* infection
Dha: 2,3-didehydroalanine
Dhb: 2,3-didehydrobutyrine
MU1140: Mutacin 1140
