# Supplementary material for: OG716: Designing a fit-for-purpose lantibiotic for the treatment of Clostridium difficile infections
Source: PLoS One. 2018 Jun 12;13(6):e0197467. doi: 10.1371/journal.pone.0197467 (PMC5997364; doi:10.1371/journal.pone.0197467)
Supplement: S1 Table — The universal one-letter code is used. Numbers indicate the residue number relative to Mutacin 1140. Ser5 is dehydrated to a Dha5 during post-translational modification. Positively charged amino-acids in green (R, H and K); negatively charged amino-acids in blue (D and E); polar amino-acids in yellow (S, T, N and Q); hydrophobic amino-acid in red (A, V, I, L, M, F, Y and W). Failed substitutions highlighted in gray. (PDF) [file pone.0197467.s001.pdf]

Clone No.	Ogxxx	F1	K2	W4	S5	L6	A12	R13	G15	Y20
1		I						N		
2	OG703	I						D		
3	OG711	I						A		
4		I						T		
5		I						K		
6		I						S		
7		I						G		
8		I						V		
9		I						I		
10		I						P		
11		I						Q		
12		I						E		
13		I						H		
14	OG712	L						N		
15		L						D		
16	OG713	L						A		
17		L						T		
18		L						K		
19		L						S		
20		L						G		
21		L						V		
22		L						I		
23		L						P		
24		L						Q		
25		L						E		
26		L						H		
27		A						N		
28	OG705	A						D		
29	OG702	A						A		
30		A						T		
31		A						K		
32		A						S		
33	OG719	A						G		
34		A						V		
35		A						I		
36		A						P		
37		A						Q		
38		A						E		
39		A						H		
40		N						N		
41		N						D		
42		N						A		
43		N						T		

44	N						K		
45	N						S		
46	N						G		
47	N						V		
48	N						I		
49	N						P		
50	N						Q		
51	N						E		
52	N						H		
53	S						N		
54	S						D		
55	S						A		
56	S						T		
57	S						K		
58	S						S		
59	S						G		
60	S						V		
61	S						I		
62	S						P		
63	S						Q		
64	S						E		
65	S						H		
66	T						N		
67	T						D		
68	T						A		
69	T						T		
70	T						K		
71	T						S		
72	T						G		
73	T						V		
74	T						I		
75	T						P		
76	T						Q		
77	T						E		
78	T						H		
79	Y						N		
80	Y						D		
81	Y						A		
82	Y						T		
83	Y						K		
84	Y						S		
85	Y						G		
86	Y						V		
87	Y						I		

88		Y						P		
89		Y						Q		
90		Y						E		
91		Y						H		
92		H						N		
93		H						D		
94		H						A		
95		H						T		
96		H						K		
97		H						S		
98		H						G		
99		H						V		
100		H						I		
101		H						P		
102		H						Q		
103		H						E		
104		H						H		
105		P						N		
106		P						D		
107		P						A		
108		P						T		
109		P						K		
110		P						S		
111		P						G		
112		P						V		
113		P						I		
114		P						P		
115		P						Q		
116		P						E		
117		P						H		
118	OG716	V						N		
119		V						D		
120		V						A		
121		V						T		
122		V						K		
123		V						S		
124		V						G		
125		V						V		
126		V						I		
127		V						P		
128		V						Q		
129		V						E		
130		V						H		
131		G						N		

132		G						D		
133		G						A		
134		G						T		
135		G						K		
136		G						S		
137		G						G		
138		G						V		
139		G						I		
140		G						P		
141		G						Q		
142		G						E		
143		G						H		
144		E						N		
145		E						D		
146		E						A		
147		E						T		
148		E						K		
149		E						S		
150		E						G		
151		E						V		
152		E						I		
153		E						P		
154		E						Q		
155		E						E		
156		E						H		
157		L						N		F
158		L						D		F
159		L						A		F
160	OG718	I						N		F
161		I						D		F
162		I						A		F
163		I	A					N		
164		I	A					D		
165		I	A					A		
166		I	A					T		
167		I	A					K		
168		I	A					S		
169		I	A					G		
170		I	A					V		
171		I	A					I		
172		I	A					P		
173		I	A					Q		
174		I	A					E		
175		I	A					H		

176	I	A					N		F
177	I	A					D		F
178	I	A					A		F
179	I	A					T		F
180	I	A					K		F
181	I	A					S		F
182	I	A					G		F
183	I	A					V		F
184	I	A					I		F
185	I	A					P		F
186	I	A					Q		F
187	I	A					E		F
188	I	A					H		F
189	I	A	K				N		
190	I	A	K				D		
191	I	A	K				A		
192	I	A	K				N		F
193	I	A	K				D		F
194	I	A	K				A		F
195	I	A	K	F			N		
196	I	A	K	F			D		
197	I	A	K	F			A		
198	I	A	K	F			N		F
199	I	A	K	F			D		F
200	I	A	K	F			A		F
201	I	T					N		
202	I	T					D		
203	I	T					A		
204	I	T					T		
205	I	T					K		
206	I	T					S		
207	I	T					G		
208	I	T					V		
209	I	T					I		
210	I	T					P		
211	I	T					Q		
212	I	T					E		
213	I	T					H		
214	I	T					N		F
215	I	T					D		F
216	I	T					A		F
217	I	T					T		F
218	I	T					K		F
219	I	T					S		F

220		I	T					G		F
221		I	T					V		F
222		I	T					I		F
223		I	T					P		F
224		I	T					Q		F
225		I	T					E		F
226		I	T					H		F
227		I	T	F	I	G		N		F
228		I	T	F	I	G		D		F
229		I	T	F	I	G		A		F
230		I	T	F	I	G	G	N		F
231		I	T	F	I	G	G	D		F
232		I	T	F	I	G	G	A		F
233		I	T	K				N		
234		I	T	K				D		
235		I	T	K				A		
236		I	T	K				N		F
237		I	T	K				D		F
238		I	T	K				A		F
239		V	T					N		
240		V	T					D		
241		V	T					A		
242		V	T					N		F
243		V	T					D		F
244		V	T					A		F
245		V	T	K				N		
246		V	T	K				D		
247		V	T	K				A		
248		V	T	K				N		F
249		V	T	K				D		F
250		V	T	K				A		F
251		I		M				N		
252		I		M				D		
253	OG708	I		M				A		
254		I		I				N		
255		I		I				D		
256		I		I				A		
257		I				V		N		
258		I				V		D		
259		I				V		A		
260		I					T	N		
261		I					T	D		
262		I					T	A		
263	OG707	I						N	A	

264	I						D	A	
265	I						A	A	
266	I	T	V				N		
267	I	T	V				D		
268	I	T	V				A		
269	I	T	M				N		
270	I	T	M				D		
271	I	T	M				A		
272	I	T	I				N		
273	I	T	I				D		
274	I	T	I				A		
275	I	T			V		N		
276	I	T			V		D		
277	I	T			V		A		
278	I	T				T	N		
279	I	T				T	D		
280	I	T				T	A		
281	I	T					N	A	
282	I	T					D	A	
283	I	T					A	A	
284	I	A	V				N		
285	I	A	V				D		
286	I	A	V				A		
287	I	A	M				N		
288	I	A	M				D		
289	I	A	M				A		
290	I	A	I				N		
291	I	A	I				D		
292	I	A	I				A		
293	I	A			V		N		
294	I	A			V		D		
295	I	A			V		A		
296	I	A				T	N		
297	I	A				T	D		
298	I	A				T	A		
299	I	A					N	A	
300	I	A					D	A	
301	I	A					A	A	
