# Supplementary material for: OG716: Designing a fit-for-purpose lantibiotic for the treatment of Clostridium difficile infections
Source: PLoS One. 2018 Jun 12;13(6):e0197467. doi: 10.1371/journal.pone.0197467 (PMC5997364; doi:10.1371/journal.pone.0197467)
Supplement: S2 Table — Top eight (8) compounds are highlighted in red. (PDF) [file pone.0197467.s002.pdf]

F1	K2	W4	S5	L6	C7	A12	R13	G15	Y20	Number of variants
Activity better than OG253 (>0.167 cm^2 zone of clearing)										
L							G			1
A							N, A, S, G, V, P			6
T							A, G, V			3
V							N[1], A, T, P, Q			5
G							N, V			2
I	A						A, T, V			3
I	A						A, G, Q		F	3
I	A	K					A			1
I	A	K					N, A		F	2
I	T	K					A		F	1
I		M					N, A			2
I		I					A			1
I							N, A	A		2
I	A						P		F	1
I	A	M					N			1
I	A	M					A			1
S	A			V			A			1
I							H			1
N							I			1
S							G			1
I	T						N			1
I	T						N	A		1
I							N, D, A, T, S, G, V, I, P, Q			10
L							N, D, A, T, S, P, Q, E			8
A							D, T, K, Q, E, H			6
N							K			1
S							N, A, T, K, V, I, Q, E			8
T							N, S, I, Q, H			5
Y							D, T, S, G, V, I, P, Q, E, H			10
H							N, A, T, K, S, V, I, Q, H			9
V							D, K, V, I, E, H			6
G							A, T, S, G, Q, H			6
L							N, D, A		F	3
I							N, D, A,		F	3
I	A						K, S, I, P, Q, H			6
I	A						N, T, K, I, E, H		F	6
I	A	K					N, D			2
I	A	K					D, T, K		F	3
I	T	K					N, A			2
I	T	K					N, D		F	2
V	T						N			1
V	T	K					A			1
V	T	K					N		F	1
I		M, I					D			2
I				V			D, A			2
I						T	N			1
I							D	A		1
I	T	V, M					N			2
I	T	M					A			1
I	T			V			A			1
I	A	V, I					A			2
I	A			V			N			1
I	A						V		F	1
I	T	V					A			1

F1	K2	W4	S5	L6	C7	A12	R13	G15	Y20	Number of variants
Activity less or similar to OG253 ($\leq$0.167 cm^2 zone of clearing)										
I							K, E			2
L							K, V, H			3
N							A, T, S, G, P Q, E			7
S							D, P			2
T							D, T			2
Y							A, K			2
H							D, G, P, E			4
P							A, T, K, S, V, I, P, E, H			9
G							D, E			2
E							N, D, A, T, K, S, G, V, I, P, E, H			12
I	A						E			1
I	A	K	F				N, D			2
I	A	K	F				A			1
I	A	K	F				N, D		F	2
I	T						D, A, T, L, S, G, V, I, P, Q, E, H			12
I	T						N, D, A, S, G, V, I, P, Q, E		F	10
I	T		F	I	G		N, D, A		F	3
I	T		F	I	G	G	N, D, A		F	3
I	T	K					D			1
V	T						D, A			2
V	T						N, D, A		F	3
V	T	K					N, D			2
V	T	K					D, A		F	2
I		I					N			1
I						T	D			1
I	T	V, M					D			2
I	T	I					N, D, A			3
I	T			V			N, D			2
I	T					T	N, D, A			3
I	T						D	A		1
I	A	V					N			1
I	A	V, M					D			2
I	A			V			D			1
I	A					T	N, D, A			3
I	A						N, D, A	A		3

F1	K2	W4	S5	L6	C7	A12	R13	G15	Y20	Number of variants
Failed to construct strain for technical or biological reasons										
L, A							I			2
N							N, D, V, H			4
S							S, H			2
T							K, P, E			3
Y							N			1
P							N, D, G, Q			4
V							S, G			2
G							K, I, P			3
E							Q			1
I	A						N, D, G			3
I	A						D, S		F	2
I	A	K	F				A		F	1
I	T						H		F	1
I				V			N			1
I						T	A			1
I	T						A	A		1
I	A	I					N, D			2
