# Supplementary material for: OG716: Designing a fit-for-purpose lantibiotic for the treatment of Clostridium difficile infections
Source: PLoS One. 2018 Jun 12;13(6):e0197467. doi: 10.1371/journal.pone.0197467 (PMC5997364; doi:10.1371/journal.pone.0197467)
Supplement: S3 Table — 1. Highlighted in green indications that the value is lower than positve control, vancomycin. 2. Highlighted in red indications that the Δ purity is lower than -10%. 3. Highlighted in red indications that the Δ concentration is less than -20%. 4. Highlighted in red indication that the solubility is below 10 mg/mL. 5. Highlighted in red indication that the half life is below 300 min. (PDF) [file pone.0197467.s003.pdf]

Compound Name	Substitution relative to MU1140	Calculated Mass (Da)	Mass by LC/MS (Da)	Minimum Inhibitory Concentration (μg/mL)[1]												
					C.difficile											
				M. luetus	ATCC 9689	ATCC BAA-1805	ATCC BAA-1875	103-1	107-1	ATCC BAA-1874	ATCC 43597	ATCC BAA-1808	Range	Mode	MIC_{50}	MIC_{90}
OG253	F1I	2230.63	2230.14	0.0625	0.5	0.5	0.25	0.06	0.25	0.25	0.25	0.25	0.06-0.5	0.25	0.25	0.5
OG702	F1A R13A	2103.44	2103.53	0.250	2	2	2	0.5	4	4	2	1	0.5-4	2	2	4
OG703	F1I R13D	2189.53	2189.34	0.0625	2	1	1	0.5	2	0.5	1	1	0.5-2	1	1	2
OG704	F1T R13G	2119.44	2120.52	1.00	8	8	8	4	16	8	8	8	4-16	8	8	16
OG705	F1L R13D	2189.53	2189.06	0.0625	1	1	1	0.5	2	1	1	1	0.5-2	1	1	2
OG706	F1I R13N	2188.55	2188.40	0.0625	0.5	0.25	0.5	0.125	1	0.5	0.25	0.5	0.125-1	0.5	0.5	1
OG707	F1I R13N G15A	2202.58	2202.61	0.250	8	8	8	2	8	8	8	8	2-8	8	8	8
OG708	F1I W4M R13A	2090.50	2090.54	0.500	8	4	4	1	4	4	4	8	1-8	4	4	8
OG711	F1I R13A	2145.52	2145.06	0.125	1	0.25	0.5	0.25	1	0.5	0.5	0.25	0.25-1	0.25	0.5	1
OG712	F1L R13N	2188.55	2188.30	0.0625	0.5	0.25	0.5	0.125	0.5	0.25	0.25	0.25	0.125-0.5	0.25	0.25	0.5
OG713	F1L R13A	2145.52	2145.05	0.125	0.5	0.25	0.5	0.25	0.5	0.5	0.5	0.5	0.25-0.5	0.5	0.5	0.5
OG715	F1S R13N	2162.47	2162.54	1.00	16	8	16	2	16	16	8	8	2-16	16	8	16
OG716	F1V R13N	2174.52	2174.40	0.0625	0.5	0.25	0.5	0.25	0.5	0.5	0.25	0.25	0.25-0.5	0.5	0.25	0.5
OG717	F1G R13N	2132.44	2132.53	1.00	8	8	8	2	16	16	8	8	2-16	8	8	16
OG718	F1I R13N Y20F	2172.55	2172.26	0.125	0.5	0.5	1	0.25	0.5	0.5	0.5	0.25	0.25-1	0.5	0.5	1
OG719	F1A R13G	2089.42	2089.52	0.500	8	8	8	2	8	8	4	8	2-8	8	8	8
				**Vancomycin control**	0.5	1	0.5	1	4	0.5	0.5	0.5	0.5-4	0.5	0.5	4

Compound Name	Forced Degradation								Solubility[4]	Half Life[5]	
	2 Days at 37°C		7 Days at 37°C		2 Days at 50°C		7 Days at 50°C		5% *D*-Mannitol (mg/mL)	FaSSGF (min)	FaSSIF (min)
	Δ Purity (%)[2]	Δ Concen (%)[3]	Δ Purity (%)	Δ Conc (%)	Δ Purity (%)	Δ Conc (%)	Δ Purity (%)	Δ Conc (%)			
OG253	-1.44	-17.6	0.54	-14.3	0.39	-13.6	-3.65	-21.5	≥ 24	>1440	14.4
OG702	-1.57	-13.3	0.60	-16.5	-0.10	-5.2	-2.25	-19.2	ND	ND	ND
OG703	-0.97	-9.2	-1.04	-13.4	-1.74	-15.0	-4.79	-30.3	7	>1440	606
OG704	0.50	-20.3	-1.90	-18.1	0.43	-18.5	-6.14	-45.8	ND	ND	ND
OG705	-0.29	-11.4	-0.61	-12.3	-1.32	-11.2	-3.22	-26.0	ND	>1440	>720
OG706	-1.15	-11.3	-6.64	-20.0	-8.05	-23.0	-21.46	-50.1	18-24	>1440	708
OG707	-0.35	-11.2	-2.39	-10.9	-3.05	-27.4	-7.73	-28.8	ND	ND	ND
OG708	-0.39	-14.2	-1.90	-22.9	0.99	-29.2	-5.03	-50.8	ND	ND	ND
OG711	-1.24	-12.5	-0.63	-10.8	-0.61	-8.7	-2.94	-40.1	12-18	>1440	>720
OG712	-0.21	-4.6	-4.63	-9.3	-3.01	-8.7	-18.06	-45.2	12	>1440	587
OG713	-0.06	-11.9	-0.56	-8.8	-1.51	-16.9	-6.98	-41.0	ND	>1440	577
OG715	1.09	-14.6	-2.08	-25.3	-3.19	-33.8	-21.26	-84.3	ND	ND	ND
OG716	-0.31	-7.4	-5.20	-14.8	-3.67	-16.9	-19.77	-47.0	12-18	>1440	552
OG717	1.12	-4.8	-2.44	-15.0	-0.01	-23.2	-17.3	-59.3	ND	ND	ND
OG718	-2.07	-9.1	-7.42	-21.0	-4.90	-29.4	-12.84	-43.8	≥ 24	>1440	>720
OG719	0.54	-7.6	1.22	-12.0	0.92	-23.6	-1.92	-31.3	ND	ND	ND
