# Supplementary figures and images for: OG716: Designing a fit-for-purpose lantibiotic for the treatment of Clostridium difficile infections
Source: PLoS One. 2018 Jun 12;13(6):e0197467. doi: 10.1371/journal.pone.0197467 (PMC5997364; doi:10.1371/journal.pone.0197467)

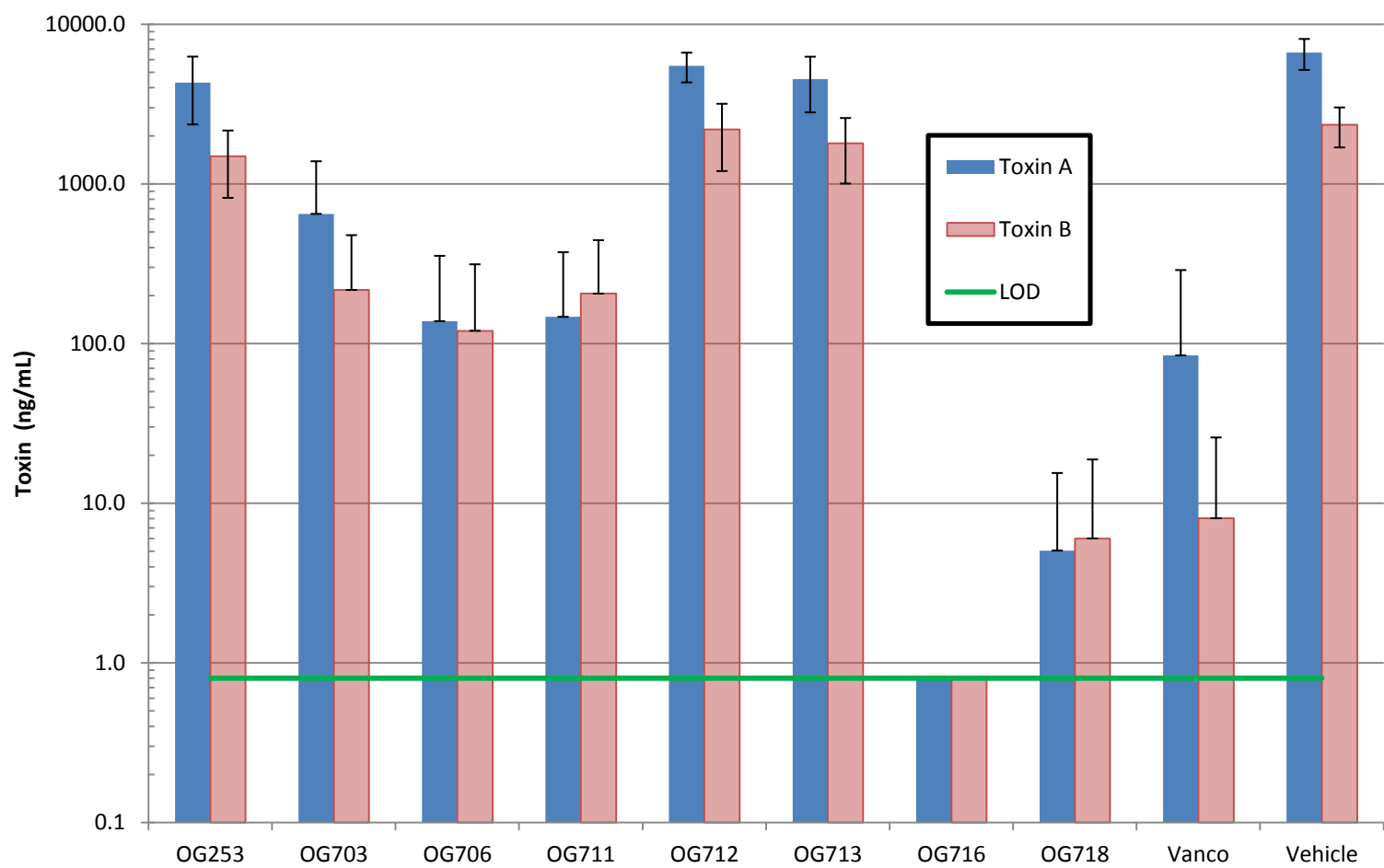

Supplement: S1 Fig — Levels of toxins A and B in the cecal contents tracked with both the measured CFU counts and survival during the study. (PDF) [file pone.0197467.s004.pdf]
